# Commentary: Are the proposed benefits of melatonin-rich foods too hard to swallow?

**DOI:** 10.3389/fnut.2016.00002

**Published:** 2016-01-25

**Authors:** Marcello Iriti, Elena Maria Varoni

**Affiliations:** ^1^Department of Agricultural and Environmental Sciences, Milan State University, Milan, Italy; ^2^Department of Biomedical, Surgical and Dental Sciences, Milan State University, Milan, Italy

**Keywords:** biokinetics, bioactive compounds, Mediterranean diet, nutraceuticals, functional food

Even if the discovery of melatonin in plants dates back to 1995 (1, 2), in the past decade, the topic of melatonin in plant foods has been largely investigated (3–5). Certainly, the detection of melatonin in grapevine (6), an important food plant, promoted the studies in this new and exciting research field. Noteworthy, it was also suggested that dietary melatonin may be regarded as a bioactive phytochemical, similar to polyphenols, as well as a component of the traditional Mediterranean diet (7–9).

In this context, the recent article by Kennaway, entitled “Are the proposed benefits of melatonin-rich foods too hard to swallow?” (10), appeared in Critical Reviews in Food Science and Nutrition, really astonished and disappointed us. In his review, the author criticized the studies investigating the measurement of blood and urine melatonin after the intake of foods containing this indoleamine. The author suggested that the claimed increases in circulating melatonin are not consistent with the amount of the dietary melatonin ingested, stating that “Studies reporting the appearance of melatonin in blood and its metabolites in urine following ingestion of melatonin-rich foods appear to be flawed.” We want to use this paper to highlight the urgency of high-quality studies on this topic, rather than to disapprove the currently available results.

Indeed, we think that some data and issues emphasized in this article can be, instead, discussed from a different perspective. Such perspective largely depends on the poor-quality of methodology applied to perform the existing studies on blood melatonin (11, 12). None of these clinical trials achieve the high standard of quality required by the evidence-based medicine approach, necessary for drug efficacy assessment as well as for phytochemical research (13). No randomized, placebo-controlled, double-blinded clinical trial is available, so far, on dietary melatonin. The use of placebo, in particular, appears to be undoubtedly one of the major problems to overcome, being that food is a very complex matrix where a plethora of active ingredients can act in concert (as melatonin and polyphenols, another important group of bioactive phytochemicals) (8).

This paucity of studies on efficacy of foods rich in melatonin largely hinders the achievement of reliable conclusions in answering the Kennaway’s question: “Are the proposed benefits of melatonin-rich foods too hard to swallow?” However, the author, lapidary, concludes: “While there may be health benefits of ingestion of certain foods it is difficult to accept that they are due to their melatonin content.” The only data used in the review to support this sentence are based on criticism of (very few) biokinetics results on blood melatonin, rather than findings on the beneficial effects of dietary melatonin on health (as mentioned before, to date still missing). It is also interesting to note as the biokinetics data on blood melatonin provided by the author as “reference” are based on studies where melatonin was administered as pure compound. It is well known, instead, how phytochemical bioavailability strictly depends on the complexity of food matrix, consisting of plant cell-wall polysaccharides, hydrosoluble and non-hydrosoluble fibers, and other inert components.

In addition, the paradigm of dietary melatonin is somewhat limiting while our knowledge on food tryptophan derivatives and indoleamines is now more and more increasing. In the last decade, our experience mainly focused on grape and wine. We demonstrated that a number of melatonin isomers are present in red wine and, probably, in other plant foods, as well as a high amount of tryptophan and tryptophan ethylester, a putative tryptophan donor (14–17). In this view, melatonin can also be produced from tryptophan locally by the gastrointestinal tract and, then, released in the bloodstream, thus confounding the levels of the food melatonin really absorbed. In fact, enteroendocrine (or enterochromaffin) cells can synthesize serotonin and melatonin from tryptophan and/or its derivatives, which are contained in the plant foods (18). Nonetheless, serotonin is abundantly contained in these foods (19) and may be directly converted into melatonin within the gut. Noteworthy, melatonin produced in the gastrointestinal tract represents a major pool of extrapineal melatonin, reaching much higher levels than in the pineal gland (20).

With regard to methodology, another hypothesis, as also suggested by the author, is that indoleamines different from melatonin, mainly its isomers, may generate misinterpretation of the results because of their cross-reactivity during the analysis. The latter has been mainly based on the enzyme-linked immunosorbent assay (ELISA), which is the sole technique, so far, used to determine blood melatonin after food intake (11, 12). We strongly encourage, in the future, the use of more reliable techniques, such as liquid chromatography coupled to mass spectroscopy (LC–MS), to assess unequivocally the amount of melatonin.

Furthermore, the production of microbial melatonin by the colonic microbiota represents another fascinating perspective to further comprehend the measured levels of melatonin after food intake. Even if no study is now available on this topic, the gut microflora may embody an alternative source of melatonin, possibly synthesized by some types of bacteria, which deserves to be investigated (20). The development of a specific LC–MS/MS method could shed significantly more light on the origin: if stable-isotope-labeled precursors are applied, the labeling pattern of melatonin and its sulfate could indicate the contribution of the different biosynthetic pathways.

We think that more scientific in human evidences should be collected before any judgment is expressed, similarly to what occurred and is still ongoing about the health-promoting effects of dietary polyphenols. We recommend more high-level studies, rather than considering “flawed” currently available data. These latter, although preliminary, can be considered a good starting point to grow knowledge on this relatively novel food component and ascertain, besides its real concentrations in blood, the effects of such levels on health.

## Author Contributions

MI and EV conceived the commentary and wrote the manuscript.

## Conflict of Interest Statement

The authors declare that the research was conducted in the absence of any commercial or financial relationships that could be construed as a potential conflict of interest.
